# Supplementary material for: Stability of clinical prediction models developed using statistical or machine learning methods
Source: Biom J. 2023 Jul 19;65(8):2200302. doi: 10.1002/bimj.202200302 (PMC10952221; doi:10.1002/bimj.202200302)
Supplement: Supplementary file 1 — Supporting Information [file BIMJ-65-0-s002.pdf]

SUPPLEMENTARY MATERIAL

Figure S1: Each plot shows instability of calibration curves for 1000 example models developed using a particular sample size (100, 200, 500, 888, 1000 or 5000) when each model is applied to the same population of 100000 individuals: each example model was produced from a logistic regression (LR) with either a full model with no penalisation (‘unshrunk LR’), a full model followed by a uniform shrinkage (‘shrunken LR’), or with a LASSO penalty fitted to a different random sample of individuals from a population with a true overall risk of 0.5, based on a true model with a linear prediction of LP = 0.5X1+0.3X2+0.3X3+0.25X4+0.25X5 + 0(Z1+…+Z10) where X1, …, X5~N(0,1) and 10 noise variables (Z1, …, Z10 ~ N(0,1)).

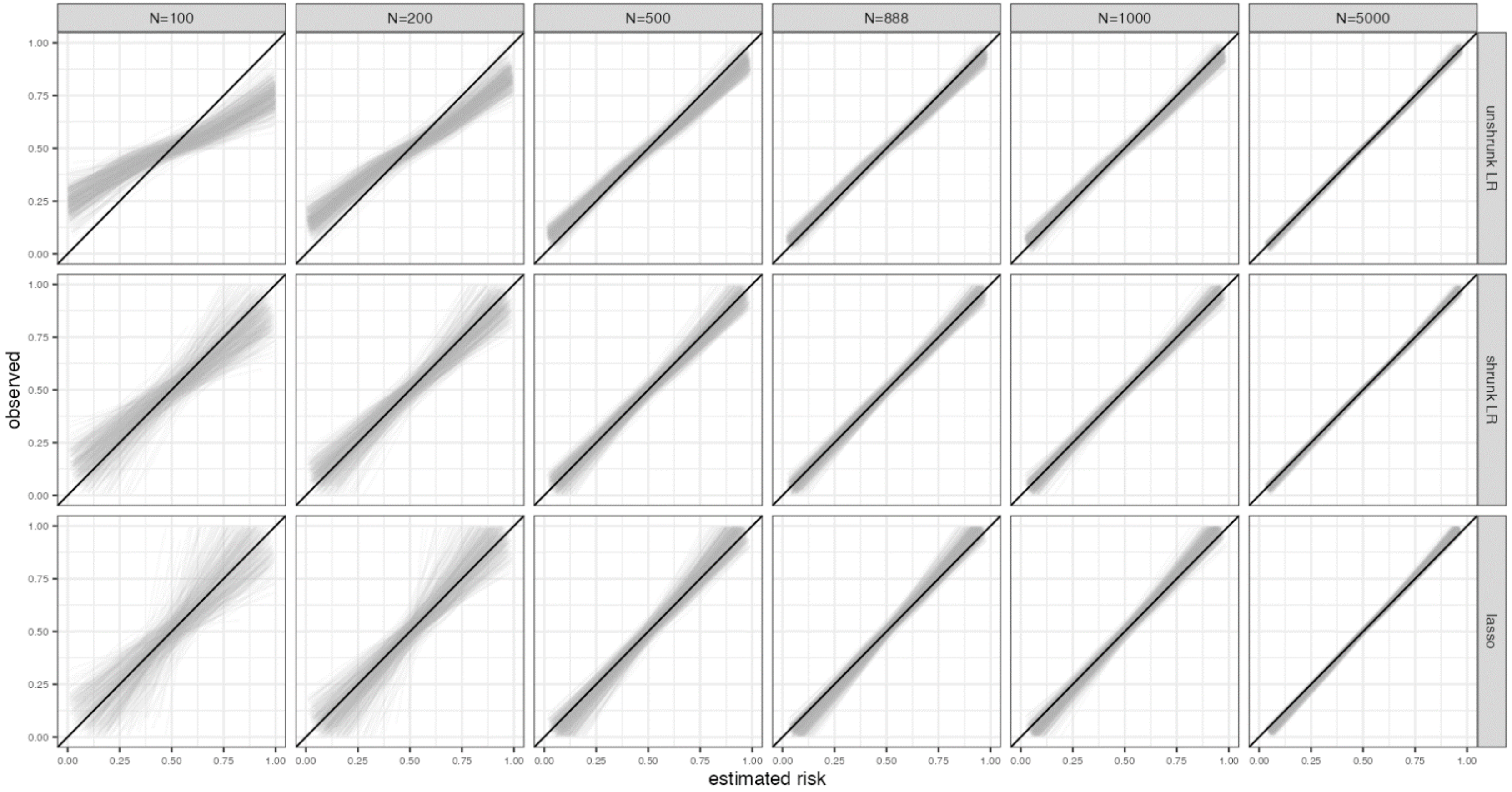

Figure S2: Instability plots and measures for three prediction models developed using 752 participants (53 events) and 7 candidate predictors (see Sections 4.3 and 4.4)

	(a) logistic regression with LASSO	(a) unpenalised logistic regression followed by uniform shrinkage of predictor effects	(c) Random forest (100 trees, unlimited depth)
**Prediction instability plot**	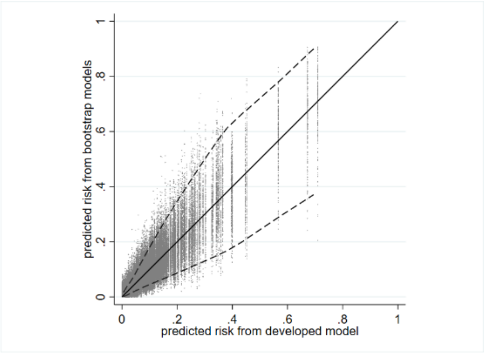	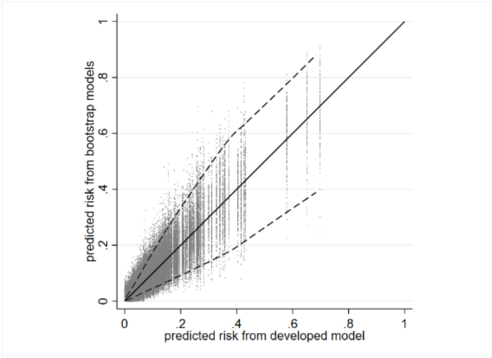	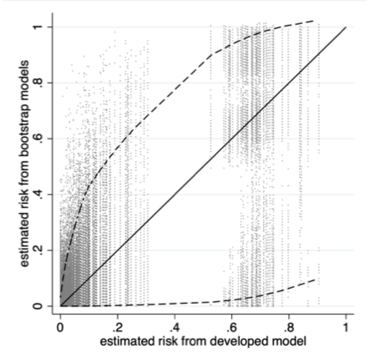
**MAPE instability plot**	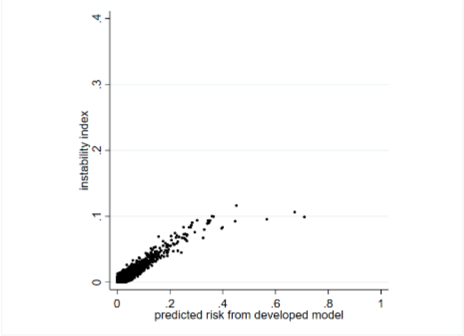	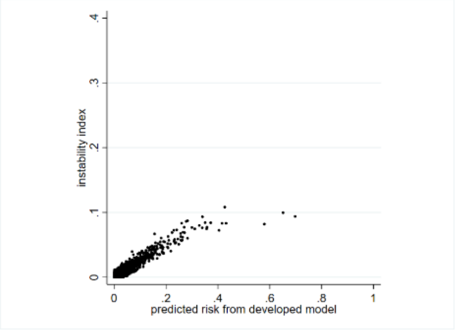	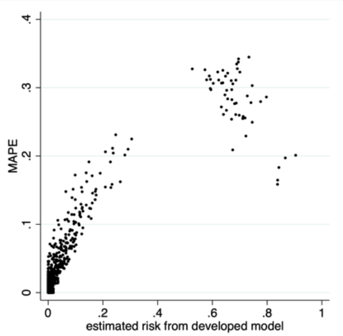
**Average MAPE**	0.019	0.018	0.047

Figure S3: Instability plots and measures for a prediction model developed using random forest (7 predictors) in 752 participants, using a variety of hyperparameter tuning options of (a) software defaults, (b) defaults but with tree depth of 3, and (c) allowing tuning to be tailored

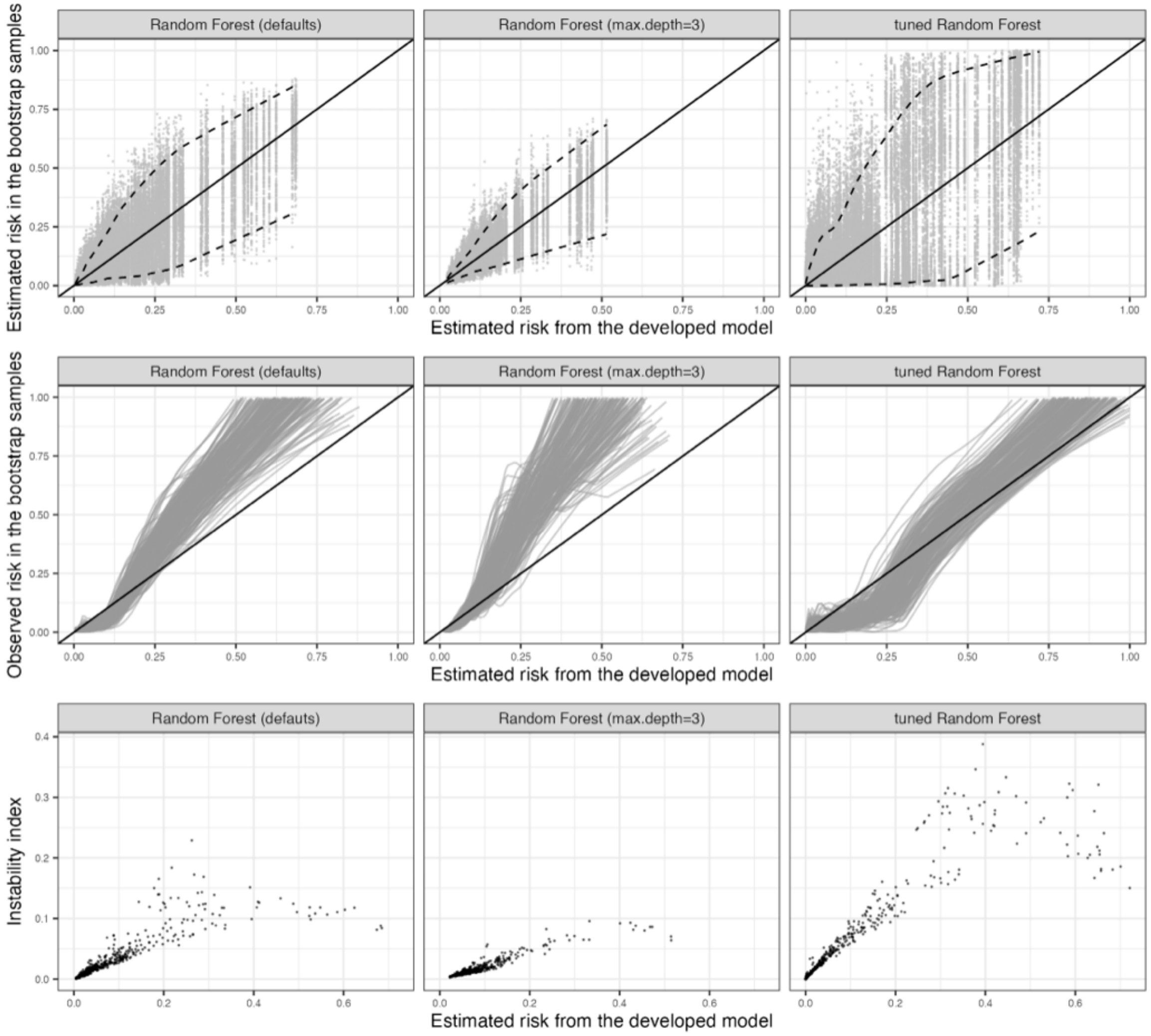

Figure S4: Instability plots and measures for a prediction model developed using random forest (7 predictors, 100 trees, depth 3) in 452 participants, followed by recalibration in 300 participants

Prediction instability plot

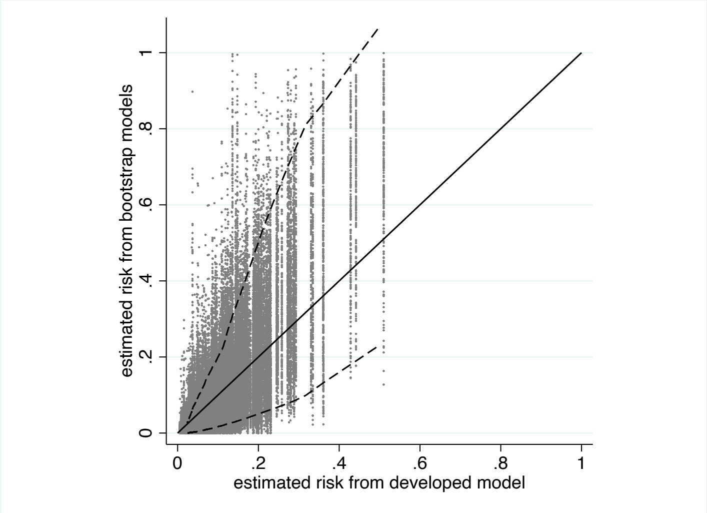

MAPE (mean 0.045)

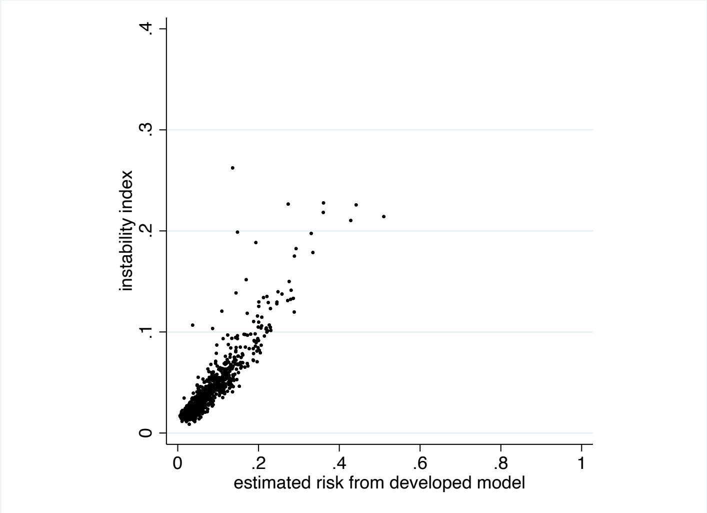

Calibration instability plot

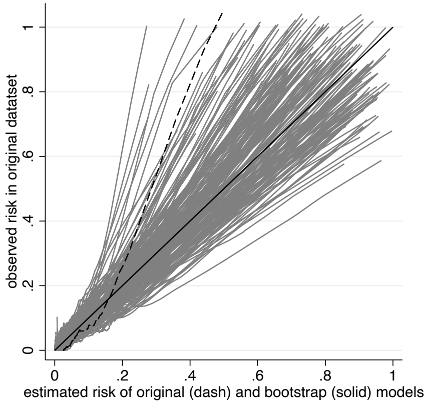

Calibration instability plot when using all 752 participants for model development without recalibration

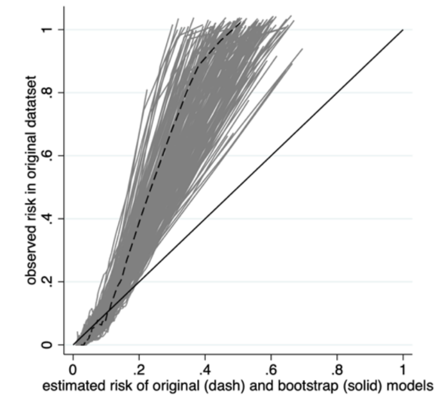

Figure S5: Instability plots and measures to examine fairness of a LASSO prediction model in males and females separately (see Section 5.1)

	Males	Females
**Prediction instability plot**	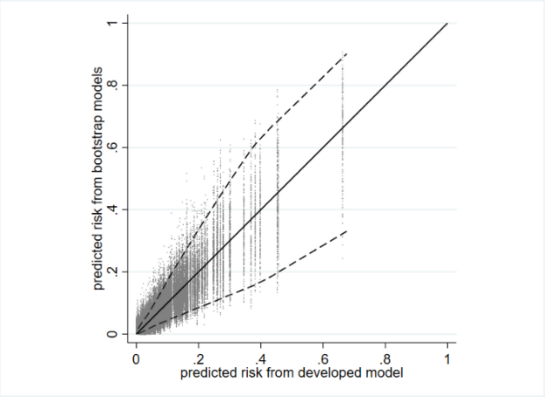	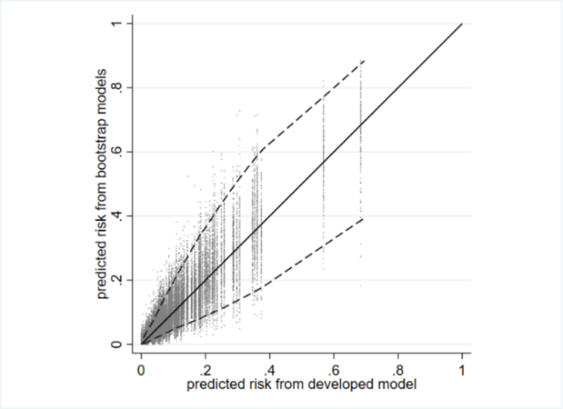
**MAPE instability plot**	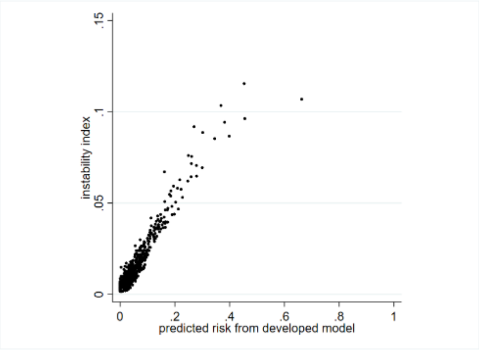	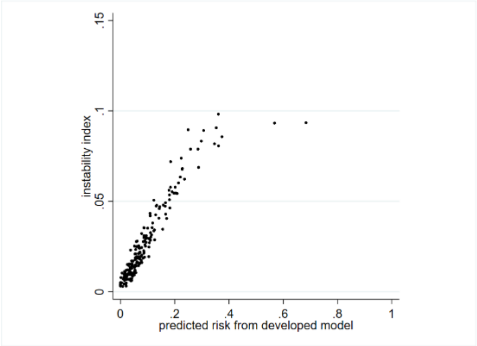
**Average MAPE**	0.017	0.027

Figure S6: Histogram of C-statistics from applying the LASSO model in Section 5.2 to the bootstrap samples

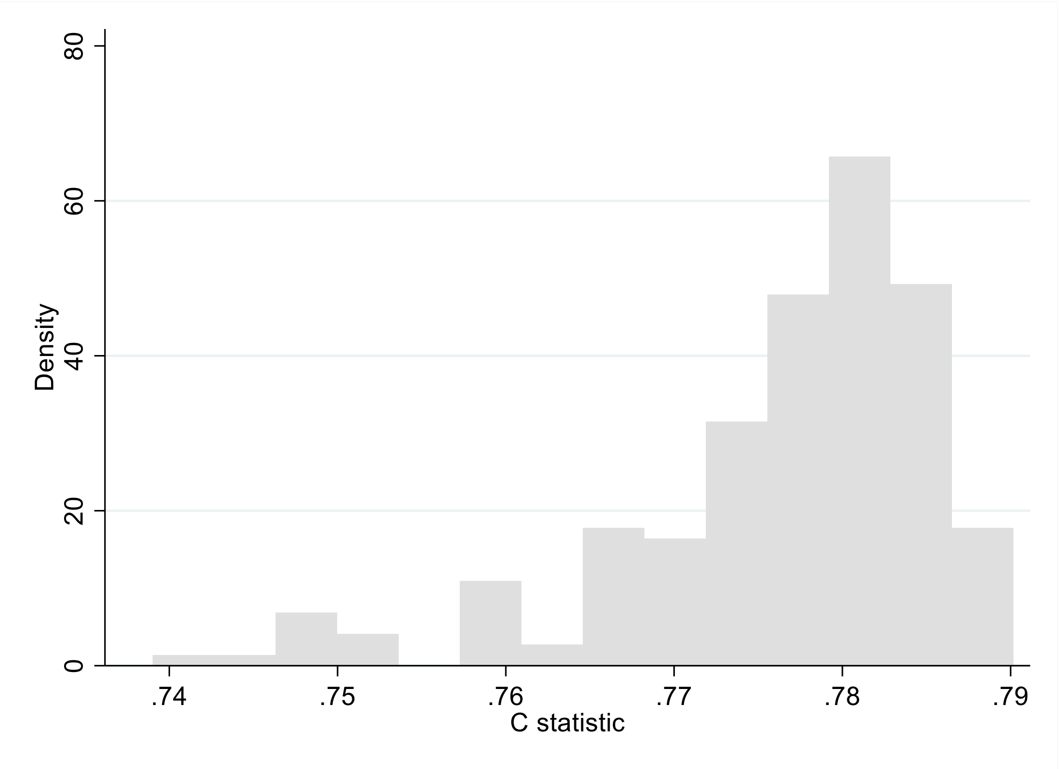

Figure S7: Classification instability plots for various models from the case studies of Section 4.

**Large sample size**<br>40830 patients, 2851 events<br>7 predictor parameters	**Minimum recommended**<br>752 patients, 53 events<br>7 predictor parameters	**Small sample size**<br>300 patients 21 events<br>7 predictor parameters
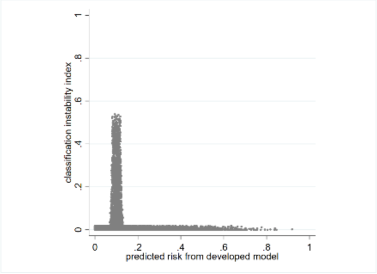	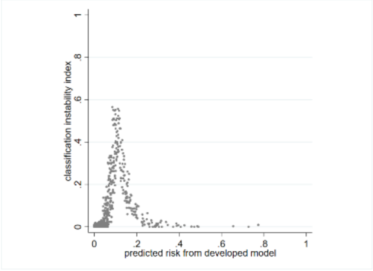	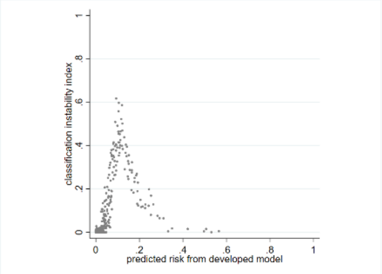
752 patients, 53 events<br>7 predictor + 20 noise<br>Force all predictors in	752 patients, 53 events<br>7 predictor + 20 noise<br>Backwards selection	752 patients, 53 events<br>7 predictor + 20 noise<br>LASSO
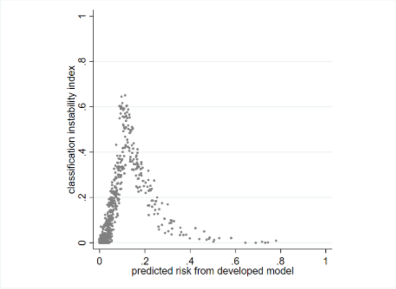	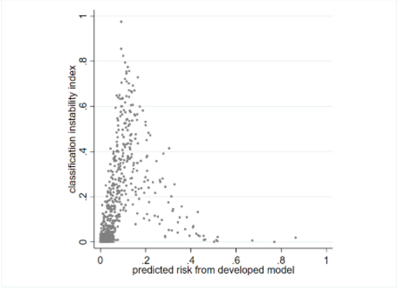	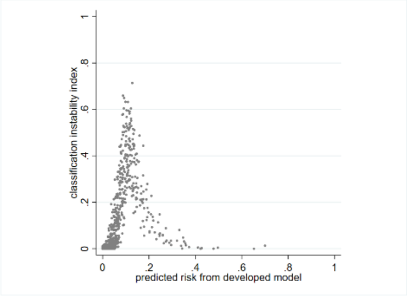
**Minimum recommended**<br>752 patients, 53 events<br>7 predictor parameters<br>LASSO	**Minimum recommended**<br>752 patients, 53 events<br>7 predictor parameters<br>Uniform	**Minimum recommended**<br>752 patients, 53 events<br>7 predictor parameters<br>Random forest
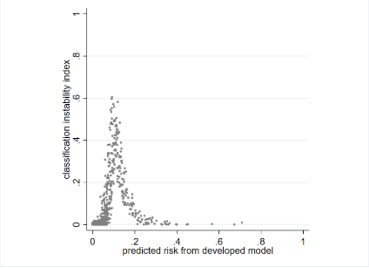	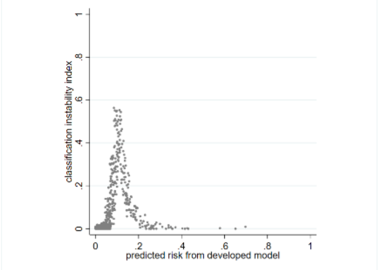	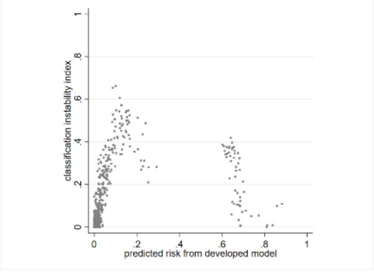

**Minimum recommended**<br>752 patients, 53 events<br>7 predictor parameters<br>LASSO - males	**Minimum recommended**<br>752 patients, 53 events<br>7 predictor parameters<br>LASSO - females	
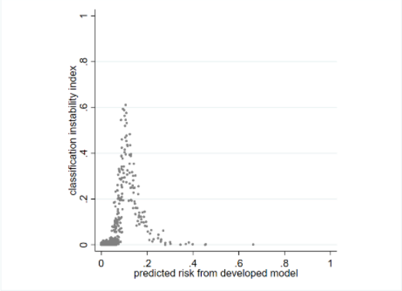	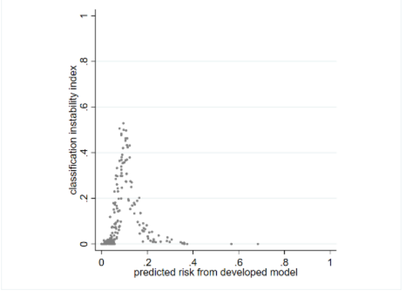	
